# Identification of epilepsy related pathways using genome-wide DNA methylation measures: A trio-based approach

**DOI:** 10.1371/journal.pone.0211917

**Published:** 2019-02-08

**Authors:** Ozkan Ozdemir, Ece Egemen, Sibel Aylin Ugur Iseri, Osman Ugur Sezerman, Nerses Bebek, Betul Baykan, Ugur Ozbek

**Affiliations:** 1 Aziz Sancar Institute of Experimental Medicine, Department of Genetics, Istanbul University, Istanbul, Turkey; 2 Department of Computer Engineering, Faculty of Engineering and Natural Sciences, Sabanci University, Istanbul, Turkey; 3 Department of Medical Statistics and Bioinformatics, School of Medicine, Acibadem Mehmet Ali Aydinlar University, Istanbul, Turkey; 4 Istanbul University, Istanbul Faculty of Medicine, Department of Neurology, Istanbul, Turkey; 5 Department of Medical Genetics, School of Medicine, Acibadem Mehmet Ali Aydinlar University, Istanbul, Turkey; Chuo University, JAPAN

## Abstract

Genetic generalized epilepsies (GGE) are genetically determined, as their name implies and they are clinically characterized by generalized seizures involving both sides of the brain in the absence of detectable brain lesions or other known causes. GGEs are yet complex and are influenced by many different genetic and environmental factors. Methylation specific epigenetic marks are one of the players of the complex epileptogenesis scenario leading to GGE. In this study, we have set out to perform genome-wide methylation profiling to analyze GGE trios each consisting of an affected parent-offspring couple along with an unaffected parent. We have developed a novel scoring scheme within trios to categorize each locus analyzed as hypo or hypermethylated. This stringent approach classified differentially methylated genes in each trio and helped us to produce trio specific and pooled gene lists with inherited and aberrant methylation levels. In order to analyze the methylation differences from a boarder perspective, we performed enrichment analysis with these lists using the PANOGA software. This collective effort has led us to detect pathways associated with the GGE phenotype, including the neurotrophin signaling pathway. We have demonstrated a trio based approach to genome-wide DNA methylation analysis that identified individual and possibly minor changes in methylation marks that could be involved in epileptogenesis leading to GGE.

## Introduction

Genetic generalized epilepsies (GGE; also known as idiopathic generalized epilepsy) comprise a distinct subgroup of epilepsy syndromes: GGEs are genetically determined and they are clinically characterized by generalized seizures involving both sides of the brain in the absence of detectable brain lesions or other known etiologies [1]. GGEs account for almost 20% of all epilepsies, which in turn affects up to 0.2% of the general population [2]. Childhood and juvenile absence epilepsy (CAE and JAE), juvenile myoclonic epilepsy (JME) and epilepsy with generalized tonic–clonic seizures (GTCS) represent the most common GGE syndromes [3].

The genetic etiology of GGE remains to be elusive for most of the cases despite presumed presence of strong genetic factors. Even when the underlying genetic aberration is known, variable expressivity and reduced penetrance are common among patients [4]. This can partly be attributed to the complex determinants of epileptogenesis leading to GGE. Aberrant epigenetic modifications have recently been emerged in various neurological diseases including epilepsy as key pathogenic or predisposing factors [5]. Epigenetics refers to a broad range of mechanisms that ultimately lead to establishment of heritable and potentially reversible chromatin marks without actual base alterations on the DNA sequence [6]. This, in turn affects every aspect of cellular and organismal plasticity and dynamics *via* spatial and temporal control of gene expression. Among all epigenetic mechanisms, DNA methylation is a well-studied modification implicated in various models of epilepsy [7]. The process involves covalent addition of a methyl group to the cytosine base of a palindromic Cytosine-phosphate-Guanine (CpG) dinucleotide to form 5-methylcytosine.

Epigenome is regulated by both inherited and environmental factors. Inherited aberrations in epigenetic marks either due to on-site sequence alterations or changes in level or activity of trans acting elements can alter the epigenome, resulting in changes at the transcriptional level [8]. Such inherited changes can be investigated in readily accessible tissues including blood, which can serve as feasible source of epigenetic biomarker detection related to epilepsy. Likewise, blood-based changes at the pathway level might point out biological processes distorted in epilepsy. We therefore set out to investigate differential DNA methylation patterns in 15 trios with inherited GGE in order to dissect the inherited alterations in blood-based methylation signatures associated with epilepsy. We then conducted gene set enrichment analysis for these trio-based changes to map pathways related to epilepsy.

## Materials and methods

### Patients and genome-wide DNA methylation measures

Genome-wide DNA methylation was profiled in whole-blood samples obtained from 15 discrete trios each having an affected child along with an affected parent. The trios had been followed up between the years 2003–2012 at Istanbul University Epilepsy Center (EPIMER). All patients underwent full clinical, neuroradiological and electroencephalography (EEG) examinations. Informed consents were obtained from all individuals in accordance with ethics approval obtained for the study from Ethics Committee of Istanbul Medical Faculty (2009/2851). Clinic information regarding the epilepsy phenotype along with age of onset data and degree of consanguinity in each trio is presented in S1 Table.

Whole-blood genomic DNA (150 ng) was treated with sodium bisulphite using the EZ DNA Methylation Gold Kit (Zymo Research, Irvine, CA), following DNA isolation performed by using QIAamp DNA maxi kit. Genome-wide DNA methylation levels were assayed in 45 samples distributed on 6 different Illumina Infinium HumanMethylation450 BeadChip (Illumina, San Diego, CA, USA), following the Illumina Infinium HD Methylation protocol. This chip covers 485,577 CpG sites per sample at single-nucleotide resolution. The CpG sites are distributed mainly within or neighboring the genes, including promoter regions each represented as two distinct blocks of 200 bp and 1500 bp upstream of the transcription start site (designated TSS200 and TSS1500, respectively) [9]. DNA methylation data produced in the study have been deposited to the NCBI GEO database (Accession Number: GSE119684).

### Data processing

Raw intensity (.idat) files were read into RnBeads package in order to perform (i) initial quality control assessments for filtering out poor-performing samples and probes, (ii) normalization and (iii) detection of batch effect for different chips. Normalization was first performed using both the *Methylumi* package for background correction and Subset-quantile Within Array Normalization (SWAN) for array normalization [10–12]. The normalized fraction of DNA methylation at a specific CpG site was calculated as beta value = M/M+U+100, where M and U are methylated and un-methylated signal intensities, respectively. The probes represented in Illumina HumanMethylation450 v1.2 manifest file was then filtered out if they had an “rs” identifier, were located on sex chromosomes, or had unreliable measurements (Greedycut algorithm; S1 Fig).

Three individuals from two trios (Trio 4 and 7) did not pass the required quality assessment due to failure in bisulphite conversion step, so these two trios were completely removed from downstream analyses leaving 13 GGE trios (S2 Fig).

### Differential methylation analysis (DMA)

Two distinct types of DMA approaches, dual and trio based, were performed in order to elucidate methylation patterns that may be associated with the GGE phenotype. In the dual approach, the data set from all affected and unaffected samples were combined respectively and compared with each other as if they were two distinct phenotype groups. Alternatively, in the trio-based approach, the data from two affected samples from one trio were combined and compared against the unaffected sample in that particular trio. The data was analyzed afterwards for the 13 trios that passed the initial quality control by RnBeads package.

#### Trio-based DMA for enrichment analysis: Implementation of PANOGA using a trio-based scoring scheme

We conducted a trio-oriented pathway approach for clustering differentially methylated genes (DMG). The whole genome beta values for each CpG site was marked as hypo- or hyper-methylated: Beta value between 0.0 and 0.2 indicates hypo-methylation and beta value between 0.8 and 1.0 indicates hyper-methylation [13]. A particular CpG site is considered to be differentially methylated, whenever there is a difference in the methylation status between the unaffected parent and the affected child-parent pair in a given trio (Table 1). This rule indicates that if the affected pair within a trio is both hypo-methylated, the unaffected parent should be hyper-methylated and *vice versa*. We first formed a list of CpG loci that fits this rule and then scored each CpG locus in this list using the scoring scheme (Trio-based scoring scheme: TbSSch) presented in Table 1. We then annotated each CpG locus using its relevant gene in order to generate 13 final lists of trio-based DMGs with our custom TbSSch. If a gene is associated with more than one marked CpG locus, the maximum of the calculated TbSSch was used in the list.

**Table 1:** Trio-based scoring scheme (TbSSch) calculations for the selected CpG loci. A beta value between 0.0 and 0.2 indicates hypo-methylation and a beta value between 0.8 and 1.0 indicates hyper-methylation.

*If methylation status of the*			*Calculate TbSSch as*	
**Child is**	**Affected Parent is**	**Unaffected Parent is**		
hyper	hyper	hypo		[(*C*^a^-0.5)+(*PA*^b^-0.5)+(0.5-*PU*^c^)]/1.5
hypo	hypo	hyper		[(0.5-*C*^a^)+(0.5-*PA*^b^)+(*PU*^c^-0.5)]/1.5

Beta values are represented respectively as C for the affected child, PA for affected parent and PU for the unaffected parent.

Additionally, a single pooled list of DMGs, namely the ‘family pool list’ has also been produced, in which genes that exist in more than one trio-based DMGs were included. The maximum TbSSch was again assigned to the family pool list for sorting purposes. The trio-based DMA analysis presented herein is also repeated for probes residing only at TSS1500 and TSS200 locations for assessing differential promoter methylation. To sum up, we have produced gene lists for 13 individual trios and a single-family pool list both for genome-wide and promoter-specific differential methylation patterns. These lists of DMGs along with their p values are given as input to PANOGA (Pathway and Network Oriented GWAS Analysis) to identify pathways with proteins that are significantly altered for each epilepsy group.

PANOGA is a protocol originally designed for identification of SNP-targeted pathways from genome-wide association (GWAS) data [14]. Herein, we have implemented PANOGA to our genome-wide DNA methylation dataset and accordingly performed enrichment analysis using genes associated with differentially methylated CpG loci instead of SNP targeted genes.

Data analyses were conducted using the default PANOGA pipeline (for details [14, 15]). Briefly, PANOGA first searches out active sub-networks containing most of the disease affected proteins in the human PPI network [16]. Active Modules algorithm [17] is employed to identify the sub-networks taking into account the P-values of each gene with the network topology to extract potentially meaningful active sub-networks that overlaps at most 50% with each other. The next step following the identification of sub-networks is to evaluate whether these sub-networks are biologically meaningful. For each sub-network, PANOGA computes the number of genes in an identified sub-network that are also found in a specific human biochemical pathway, compared to the overall number of genes described for that pathway. In this functional enrichment step, PANOGA uses a two-sided (Enrichment/Depletion) test based on the hyper-geometric distribution to examine the association between the genes introduced to PANOGA and the genes found in each Kyoto Encyclopedia of Genes and Genomes (KEGG) pathway [18]. To correct the p-values for multiple testing, the Bonferroni correction procedure was applied on the p-values of each identified pathway. If a KEGG pathway is determined statistically significant for at least one of the active sub-networks, PANOGA adds this pathway into our final list of significant KEGG pathways associated with disease. If a pathway appears in more than one subnetwork analysis only the most significant one is reported.

#### Dual DMA: Comparison of the epileptic versus healthy groups for KEGG annotation

The dual DMA approach focuses on identification of any possible methylation mark specific to the pooled epileptic group using the pooled healthy group as a control. For this analysis, initially DNA methylation heatmap was constructed using unsupervised hierarchical clustering of CpG sites with the highest variance in methylation across all samples. Then the correlation in average methylation values for epileptic versus healthy group was plotted were plotted.

We then used the average beta values values in order to annotate KEGG pathways identified via the PANOGA analysis. For the the average beta values for epileptic and healthy groups were listed for island probes. The probes were mapped to relevant genes and only-pathway related genes were sorted. The average beta value for each gene for epileptic and healthy groups were rescaled into a range between -1 and 1 using the function: y = C+(x-A)(D-C)/(B-A), where A and B stands for minimum and maximum points of the data; C and D equals to the extreme points of the new scale. x is the related value in our data set and y is the new scaled value. Since beta values range from 0 to 1, we used a larger range to scale beta values from 0 to 0.5 into -1 to 0 and 0.5 to 1 into 0 to 1, in order to get a clearer understanding for genes involved in KEGG pathway. Eventually, these values were integrated into KEGG pathway via the Pathview package, which is an R/Bioconductor package for pathway-based data integration and visualization [19]. If a gene has a single color, then the methylation rage is the same both across epileptic and healthy groups. If the gene has a dual color, then the left side represents the methylation status for epileptic and the right side represents the healthy group.

## Results

**Fig 1:**
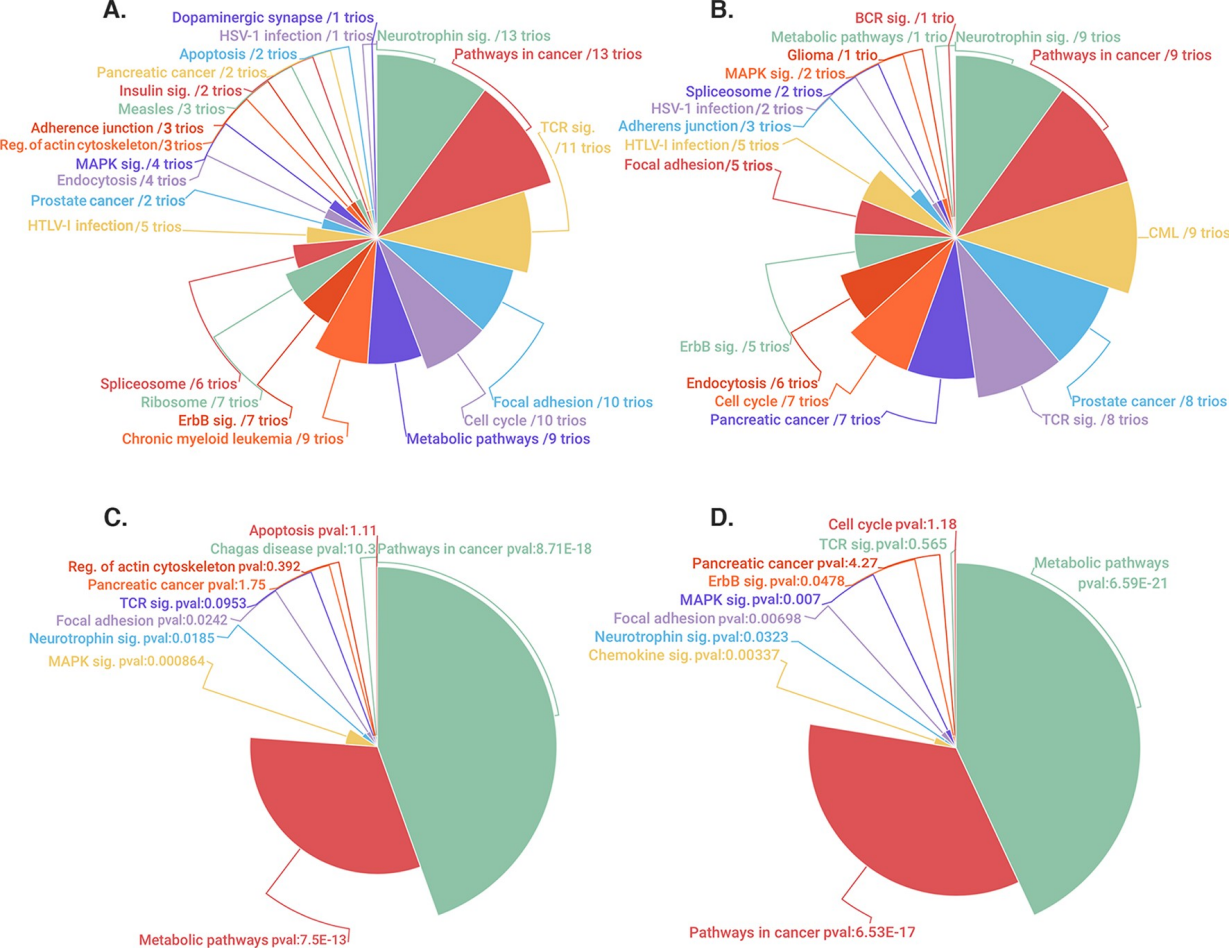
Mapping of TbSSch selected genes to relevant pathways by PANOGA. The pie chats illustrate the distinct pathways identified using trio based and family pool gene lists. (**A-B):** The trio-based pathways are shown both for genome-wide and promoter specific analyses along with the number of trios, in which that particular pathway was identified. **C-D:** The most significant 10 pathways identified through the family-pool analysis are shown both for genome-wide and promoter specific analyses along with their p-values.

Herein we present pathway oriented genome-wide DNA methylation analyses related to 13 GGE trios which meet relevant quality control metrics (S1 Table). We have focused on a novel enrichment analysis strategy by using a custom designed TbSSch for each CpG locus in individual trios. Stringent cut-off values for hypomethylation (0–0.2) and hypermethylation (0.8–1) were set initially and differentially methylated CpGs were selected in each trio if this locus was hypomethylated in the affected parent-child couple compared to the unaffected parent or *vice versa*. In this context, analyses were made for 13 trios and family pools according to genome-wide and promoter site CpG loci. The relevant loci were mapped to associated genes and imputed with PANOGA protocol to analyze pathway specific variances. The statistically significant pathways found in the analysis are given in Fig 1. Details about the analysis are also given in S2–S5 Tables.

**Fig 2:**
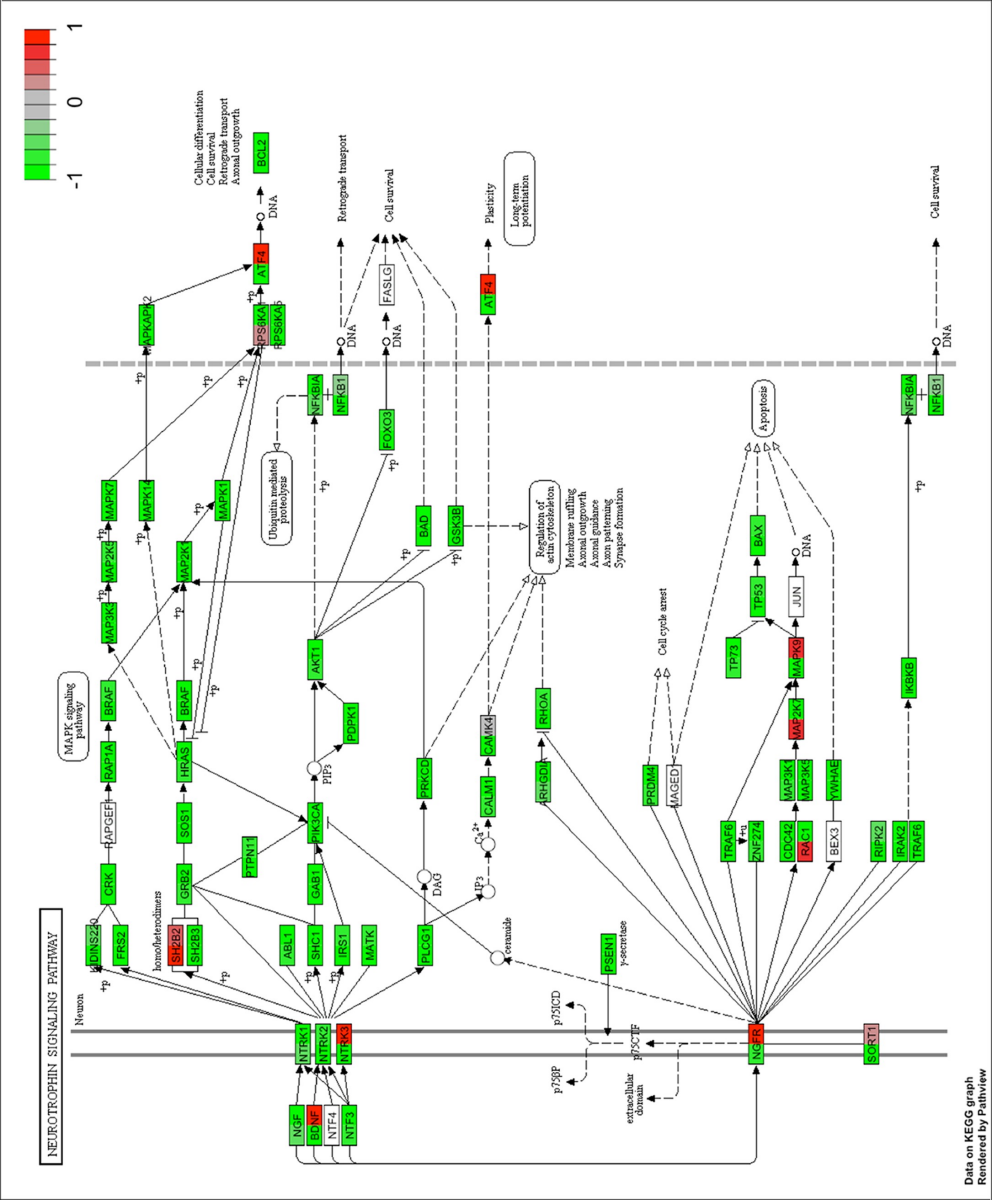
PANOGA identifies the neurotrophin pathway using the genome-wide DNA methylation dataset. The genes in the pathway are dual-colored relative to epileptic and healthy groups. Rescaled b values are colored from green to red using the new (-1, 1) range. Epileptic group is represented on the left half and healthy group on the right half of the box representing each gene.

**Fig 3:**
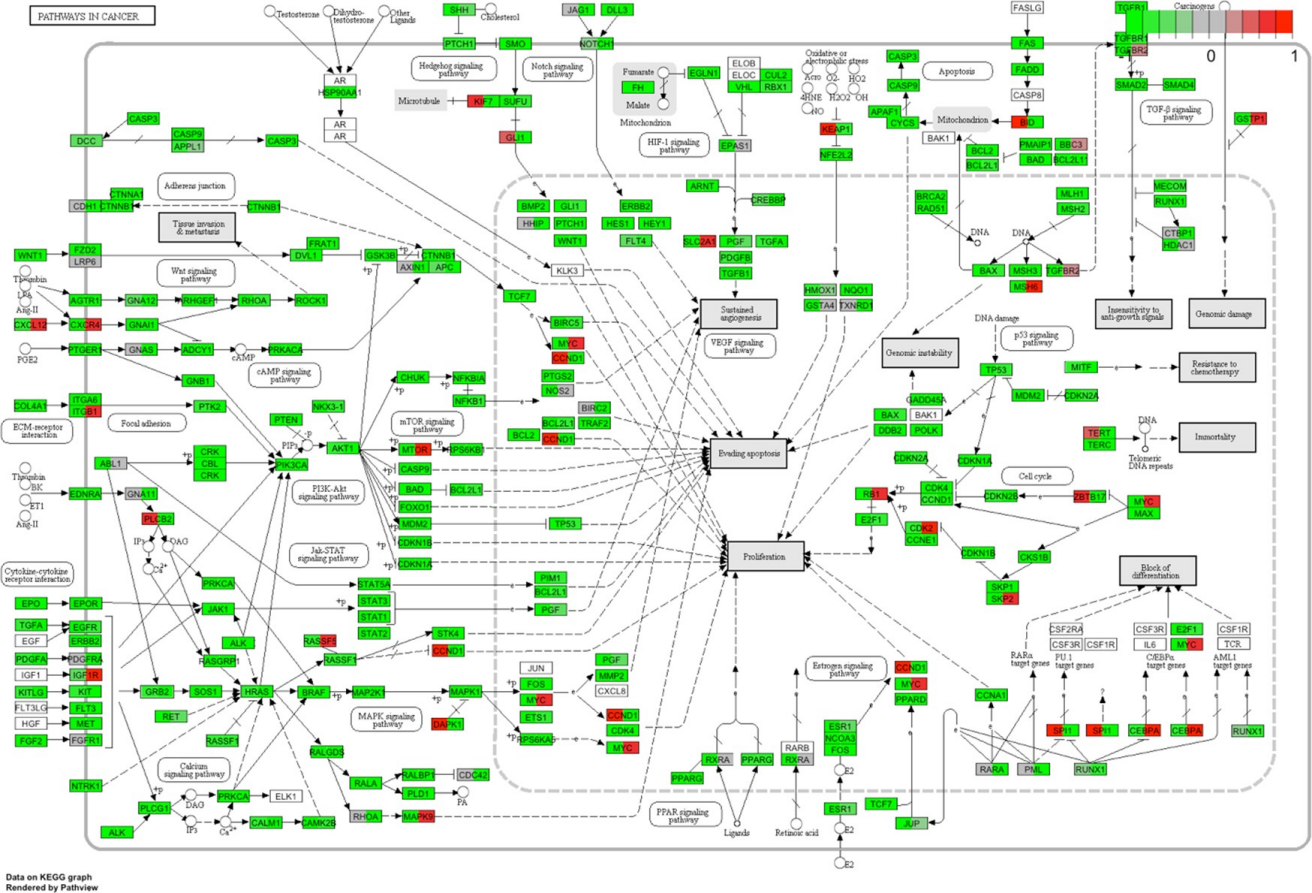
PANOGA identifies the pathways in cancer using the genome-wide DNA methylation dataset. The genes in the pathway are dual-colored relative to epileptic and healthy groups. Rescaled b values are colored from green to red using the new (-1, 1) range. Epileptic group is represented on the left half and healthy group on the right half of the box representing each gene.

Fig 1 shows PANOGA detected pathways in 13 trios and family pool both in the genome-wide and promoter level analysis. Among these pathways, ‘Neurotrophin Signaling Pathway’, ‘Pathways in Cancer’, ‘Focal Adhesion’ and ‘Metabolic Pathway’ reside both in genome level and promoter specific trio lists and also found to be significant with a p value of smaller than 0.05 in family pool analyses. The neurotrophin and cancer signaling pathways are the most common pathways; they are detected in all trios analyzed at the genome level. Neurotrophin signaling pathway appears to be the most epilepsy related pathway. The genes residing in these two KEGG pathways are dual colored according to rescaled average beta values from island-specific probes in epileptic and healthy groups (Figs 2 and 3). The list of dual colored and thus differentially methylated genes between epileptic and healthy groups for neurotrophin and cancer signaling pathways are tabulated in S6 Table along with their description and associated phenotypes (ENSEMBL-Biomart). The usual suspects for epilepsy, namely brain derived neurotrophic factor (*BDNF*) detected from the neurotrophin and solute carrier family 2 member 1 (*SLC2A1/GLUT1*) from the cancer pathway are highlighted *via* this approach. These genes along with other differentially methylated genes may be biomarkers for epilepsy. KEGG pathway sketches with dual gene coloring for other pathways are given in S4–S8 Figs.

## Discussion

In this study we have analyzed genome-wide DNA methylation profiles, of epileptic and healthy individuals, derived from vertically inherited parent-offspring trios.

In biologically unsupervised analyzes, the phenotypic effects created by genes working together are overlooked. For this reason, some genes in the genome-wide DNA methylation data set may escape identification with the DMA algorithms. To address this problem, gene lists for all thirteen families and the family pool were run through the PANOGA implementation separately. The aim of this analysis was to investigate the common pathways for scored and filtered genes. ‘Neurotrophin Signaling Pathway’, ‘Pathways in Cancer’, ‘Focal Adhesion’ and ‘Metabolic Pathway’ were all identified in trio-based enrichment analysis and they were also significant in the family pool analysis (Fig 1). The neurotrophin signaling pathway was one of the most common pathways identified, which was found in all 13 trios with high significance.

Neurotrophins define a set of molecules, which are responsible for differentiation and survival of neural cells and their relationship with epilepsy were shown in many studies [20–22]. KEGG annotation of the methylation profiles produced a set of genes guided by functional information. Shetty et al. emphasized the positive correlation between increased "nerve growth factor" (NGF) expression and dentate mossy fiber sprouting that has been associated with mesial temporal lobe epilepsy. Moreover Adams et al. reported that the NGF blockage inhibited the seizure activity with mossy fiber sprouting in kindling model of epilepsy [21–23]. Our findings showed decreased NGF methylation level with a group of neurotropic factor molecules (Figs 2 and 3). With the differential methylation analysis alone, it could be challenging to split up these findings from genome-wide DNA methylation data without pathway-oriented approach. Therefore, it is important to investigate the importance to the genes that were found differentially methylated in pathways via the PANOGA in future studies (S4–S8 Fig).

‘Pathways in cancer’ is a constellation of many smaller pathways and gene cascades implemented in DNA damage, proliferation, angiogenesis and apoptosis [24]. Therefore, it may be important to consider the breakdown of this pathway and functionally analyze the resulting sub-pathways and associated genes. Fig 1 illustrates the sub-pathways associated with huge cancer pathway, including focal adhesion and several signaling pathways such as mTOR, PPAR and p53.

In this study, we have used a novel approach for clustering genome wide differentially methylated regions using trio datasets. Differentially methylated DNA are one of the players in the complex scenario leading to GGE phenotype. Therefore, it is hard to associate gene specific minor DNA methylation variances with epilepsy. We used an adapted PANOGA approach, where genes with minor differences in DNA methylation were enriched for epilepsy related pathway detection. The trios were pivotal for this analysis; as very stringent cut-off values could be set for each CpG locus analyzed in a given trio. The inherited DNA methylation marks within each trio were analyzed in the pathway level, which are potential targets for epilepsy research and therapy. It is also important to evaluate genes and pathways in terms of expression and common genetic variants as well as DNA methylation marks.

## Supporting information

S1 Table Clinical features of father-mother-offspring trios. CAE; childhood absence epilepsy, EEG; electroencephalography, F; female, GGE; Genetic generalized epilepsy, GSWD; generalized spike and wave discharges, GTCS; generalized tonic-clonic seizure, IPS; intermittent photic stimulation, JAE; juvenile absence epilepsy, JME; juvenile myoclonic epilepsy, M; male, myo; myoclonia, N; normal, NSTP; nonspecific theta paroxysms, SE; status epilepticus, TV; television, y; year, mo; month old, AoO; age of onset, EMA; eyelid myoclonia with absences.
(DOCX)

S2 Table The most significant 10 pathways identified through the genome-wide family-pool analysis. (DOCX)

S3 Table Trio-based pathways for genome-wide analysis along with the number of trios, in which that particular pathway was identified. (DOCX)

S4 Table The most significant 10 pathways identified through the promoter specific family-pool analysis. (DOCX)

S5 Table Trio-based pathways for promoter specific analysis along with the number of trios, in which that particular pathway was identified. (DOCX)

S6 Table List of differentially methylated genes within neurotrophin and cancer pathways along with their description and associated phenotypes. The data is exported *via* BioMart tool of ENSEMBL.
(XLS)

S1 Fig Final outcome of probe and quality filtering steps. 25263 probes and 3 samples were removed from further analyses.
(TIF)

S2 Fig Control of bisulphite conversion with RnBeads package. The samples indicated by the blue arrow are 6929726053_R05C01, 6929726054_R01C02 and 6929726054_R03C02 in Trio4 and Trio7, respectively.
(TIF)

S3 Fig Differential methylation analysis via T-test. **(A)** Unsupervised hierarchical clustering of healthy (orange) and epileptic (green) individuals for all probes based on their beta values. The heatmap displays only CpG islands with the highest variance across all samples. Red color indicates beta values in the range of 0–0.5 (hypomethylation); blue color indicates beta values between 0.5–1 (hypermethylation) **(B)** Scatter plot for differential methylation analysis through T-test. The colored points (blue/red) represent differentially methylated sites according to FDR (Benjamini-Hochberg) adjustment which was set at 0.05 (None of the probes found as significant with T-test analysis after FDR adjustment, therefore there isn’t any red point on the graph). If differential methylation is not colored according to a gradient criterion, brighter colors correspond to higher point density.
(TIF)

S4 Fig PANOGA identified the KEGG metabolic pathway from trio based genome-wide DNA methylation dataset. The pathway is dual-colored on the gene level using epileptic and healthy groups. Rescaled beta values are colored from green to red using the new (-1, 1) range. Epileptic group is represented on the left half and healthy group on the right half of the box representing each gene.
(TIF)

S5 Fig PANOGA identified the KEGG focal adhesion pathway from trio based genome-wide DNA methylation dataset. The pathway is dual-colored on the gene level using epileptic and healthy groups. Rescaled beta values are colored from green to red using the new (-1, 1) range. Epileptic group is represented on the left half and healthy group on the right half of the box representing each gene.
(TIF)

S6 Fig PANOGA identified the KEGG ‘MAPK Signaling pathway’ from trio based genome-wide DNA methylation dataset. The pathway is dual-colored on the gene level using epileptic and healthy groups. Rescaled beta values are colored from green to red using the new (-1, 1) range. Epileptic group is represented on the left half and healthy group on the right half of the box representing each gene.
(TIF)

S7 Fig PANOGA identified the KEGG ‘T Cell Receptor (TCR) Signaling Pathway’ from trio based genome-wide DNA methylation dataset. The pathway is dual-colored on the gene level using epileptic and healthy groups. Rescaled beta values are colored from green to red using the new (-1, 1) range. Epileptic group is represented on the left half and healthy group on the right half of the box representing each gene.
(TIF)

S8 Fig PANOGA identified the KEGG ‘Chronic Myeloid Leukemia (CML) Pathway’ from trio based genome-wide DNA methylation dataset. The pathway is dual-colored on the gene level using epileptic and healthy groups. Rescaled beta values are colored from green to red using the new (-1, 1) range. Epileptic group is represented on the left half and healthy group on the right half of the box representing each gene.
(TIF)
