# Supplementary material for: Identification of epilepsy related pathways using genome-wide DNA methylation measures: A trio-based approach
Source: PLoS One. 2019 Feb 8;14(2):e0211917. doi: 10.1371/journal.pone.0211917 (PMC6368378; doi:10.1371/journal.pone.0211917)
Supplement: S1 Table — CAE; childhood absence epilepsy, EEG; electroencephalography, F; female, GGE; Genetic generalized epilepsy, GSWD; generalized spike and wave discharges, GTCS; generalized tonic-clonic seizure, IPS; intermittent photic stimulation, JAE; juvenile absence epilepsy, JME; juvenile myoclonic epilepsy, M; male, myo; myoclonia, N; normal, NSTP; nonspecific theta paroxysms, SE; status epilepticus, TV; television, y; year, mo; month old, AoO; age of onset, EMA; eyelid myoclonia with absences. (DOCX) [file pone.0211917.s001.docx]

**S1 Table: Clinical features of father-mother-offspring trios.** CAE; childhood absence epilepsy, EEG; electroencephalography, F; female, GGE; Genetic generalized epilepsy, GSWD; generalized spike and wave discharges, GTCS; generalized tonic-clonic seizure, IPS; intermittent photic stimulation, JAE; juvenile absence epilepsy, JME; juvenile myoclonic epilepsy, M; male, myo; myoclonia, N; normal, NSTP; nonspecific theta paroxysms, SE; status epilepticus, TV; television, y; year, mo; month old, AoO; age of onset, EMA; eyelid myoclonia with absences.

| **Trio No** | **Family relation** | **Consanguinity** | **Clinics** |
| --- | --- | --- | --- |
| Trio1 | Father | - | Myo (AoO: 20y); GTCS (AoO:19y ) |
|  | Mother |  | not affected |
|  | Daughter |  | Myo (AoO: 15 y), GTCS (AoO:16 y) |
| Trio2 | Father | + | not affected |
|  | Mother |  | JME: Myo and GTCS (AoO: 14y) |
|  | Daughter |  | JME: Myo and GTCS (AoO: 12y) |
| Trio3 | Father | + | not affected |
|  | Mother |  | Absences (10 to 20y) |
|  | Daughter |  | Absences (AoO:12y), GTCS (AoO: 15y) |
| Trio4 | Father | - | not affected |
|  | Mother |  | JME: Myo between 10-17 y |
|  | Son |  | JME: : Myo and GTCS (AoO: 12y) |
| Trio5 | Father | - | not affected |
|  | Mother |  | GTCS (AoO: 14y), myo (21y) |
|  | Son |  | Absences (AoO:14y), GTCS (AoO: 14y), myo (14y) |
| Trio6 | Father | - | not affected |
|  | Mother |  | GGE (absences, GTCS) |
|  | Son |  | GGE (absences, GTCS ) |
| Trio7 | Father | + | Absences (AoO: 8y), single GTCS at 24y of age |
|  | Mother |  | not affected |
|  | Daughter |  | Absences (AoO: 8y), GTCS (12y) |
| Trio8 | Father | + | GTCS (AoO: 15 y), myo(AoO: 15 y) |
|  | Mother |  | not affected |
|  | Daughter |  | GTCS (AoO: 17 y), myo(AoO: 20 y) |
| Trio9 | Father | - | GGE (GTCS, AoO: 20y) |
|  | Mother |  | not affected |
|  | Daughter |  | JME: Myo and GTCS (AoO: 15 y), febrile seizure history |
| Trio10 | Father | - | not affected |
|  | Mother |  | JME [Myo and GTCS (AoO:14y, Febrile seizure (7mo)) |
|  | Daughter |  | JME (Absences ( AoO: 6y, myo (AoO: 10y] |
| Trio11 | Father | + | Absences (AoO: 8y), single GTCS at 24y of age |
|  | Mother |  | not affected |
|  | Daughter |  | Absences (AoO: 8y), GTCS (12y) |
| Trio12 | Father | - | not affected |
|  | Mother |  | GGE, GTCS |
|  | Son |  | GGE, GTCS |
| Trio13 | Father | + | not affected |
|  | Mother |  | JK, GGE |
|  | Son |  | GGE |
| Trio14 | Father | - | not affected |
|  | Mother |  | GGE, GTCS |
|  | Daughter |  | JK, Absence |
| Trio15 | Father | + | not affected |
|  | Mother |  | JK, Myoclonia, Absence (EMA) |
|  | Son |  | JK, Myoclonia, Absence (EMA) |
