# Supplementary material for: Identification of epilepsy related pathways using genome-wide DNA methylation measures: A trio-based approach
Source: PLoS One. 2019 Feb 8;14(2):e0211917. doi: 10.1371/journal.pone.0211917 (PMC6368378; doi:10.1371/journal.pone.0211917)
Supplement: S2 Table — (DOCX) [file pone.0211917.s002.docx]

**S2 Table.** **The most significant 10 pathways identified through the genome-wide family-pool analysis**.

| KEGG ID | KEGG Term | p-value |
| --- | --- | --- |
| KEGG:05200 | Pathways in cancer | 8.71E-18 |
| KEGG:01100 | Metabolic pathways | 7.50E-13 |
| KEGG:04010 | MAPK signalling pathway | 8.64E-04 |
| KEGG:04722 | Neurotrophin signalling pathway | 1.85E-02 |
| KEGG:04510 | Focal adhesion | 2.42E-02 |
| KEGG:04660 | T cell receptor signalling pathway | 9.53E-02 |
| KEGG:05212 | Pancreatic cancer | 1.75E+00 |
| KEGG:04810 | Regulation of actin cytoskeleton | 3.92E-01 |
| KEGG:05142 | Chagas disease (American trypanosomiasis) | 1.03E+01 |
| KEGG:04210 | Apoptosis | 1.11E+00 |
