# Supplementary material for: Identification of epilepsy related pathways using genome-wide DNA methylation measures: A trio-based approach
Source: PLoS One. 2019 Feb 8;14(2):e0211917. doi: 10.1371/journal.pone.0211917 (PMC6368378; doi:10.1371/journal.pone.0211917)
Supplement: S3 Table — (DOCX) [file pone.0211917.s003.docx]

**S3 Table. Trio-based pathways for genome-wide analysis along with the number of trios, in which that particular pathway was identified.**

| Pathway | Count |
| --- | --- |
| Neurotrophin signaling pathway | 13 |
| Pathways in cancer | 13 |
| T cell receptor signaling pathway | 11 |
| Focal adhesion | 10 |
| Cell cycle | 10 |
| Metabolic pathways | 9 |
| Chronic myeloid leukemia | 9 |
| ErbB signaling pathway | 7 |
| Ribosome | 7 |
| Spliceosome | 6 |
| HTLV-I infection | 5 |
| Prostate cancer | 4 |
| Endocytosis | 4 |
| MAPK signaling pathway | 4 |
| Regulation of actin cytoskeleton | 3 |
| Adherence junction | 3 |
| Measles | 3 |
| Insulin signaling pathway | 2 |
| Pancreatic cancer | 2 |
| Apoptosis | 2 |
| Herpes simplex infection | 1 |
| Dopaminergic synapse | 1 |
