# Supplementary material for: Identification of epilepsy related pathways using genome-wide DNA methylation measures: A trio-based approach
Source: PLoS One. 2019 Feb 8;14(2):e0211917. doi: 10.1371/journal.pone.0211917 (PMC6368378; doi:10.1371/journal.pone.0211917)
Supplement: S4 Table — (DOCX) [file pone.0211917.s004.docx]

**S4 Table. The most significant 10 pathways identified through the promoter specific family-pool analysis**.

| KEGG ID | KEGG Term | p-value |
| --- | --- | --- |
| KEGG:01100 | Metabolic pathways | 6.59E-21 |
| KEGG:05200 | Pathways in cancer | 6.53E-17 |
| KEGG:04062 | Chemokine signalling pathway | 3.37E-03 |
| KEGG:04722 | Neurotrophin signalling pathway | 3.23E-02 |
| KEGG:04510 | Focal adhesion | 6.98E-03 |
| KEGG:04010 | MAPK signalling pathway | 7.39E-03 |
| KEGG:04012 | ErbB signalling pathway | 4.78E-02 |
| KEGG:05212 | Pancreatic cancer | 4.27E+00 |
| KEGG:04660 | T cell receptor signalling pathway | 5.65E-01 |
| KEGG:04110 | Cell cycle | 1.18E+00 |
