# Supplementary material for: Identification of epilepsy related pathways using genome-wide DNA methylation measures: A trio-based approach
Source: PLoS One. 2019 Feb 8;14(2):e0211917. doi: 10.1371/journal.pone.0211917 (PMC6368378; doi:10.1371/journal.pone.0211917)
Supplement: S5 Table — (DOCX) [file pone.0211917.s005.docx]

**S5 Table.  Trio-based pathways for promoter specific analysis along with the number of trios, in which that particular pathway was identified.**

| Pathway | Count |
| --- | --- |
| Neurotrophin signaling pathway | 9 |
| Pathways in cancer | 9 |
| Chronic myeloid leukemia | 9 |
| Prostate cancer | 8 |
| T cell receptor signaling pathway | 8 |
| Pancreatic cancer | 7 |
| Cell cycle | 7 |
| Endocytosis | 6 |
| ErbB signaling pathway | 5 |
| Focal adhesion | 5 |
| HTLV-I infection | 5 |
| Adherens junction | 3 |
| Herpes simplex infection | 2 |
| Spliceosome | 2 |
| MAPK signaling pathway | 2 |
| Glioma | 1 |
| Metabolic pathways | 1 |
| B cell receptor signaling pathway | 1 |
