# Supplementary figures and images for: Identification of epilepsy related pathways using genome-wide DNA methylation measures: A trio-based approach
Source: PLoS One. 2019 Feb 8;14(2):e0211917. doi: 10.1371/journal.pone.0211917 (PMC6368378; doi:10.1371/journal.pone.0211917)

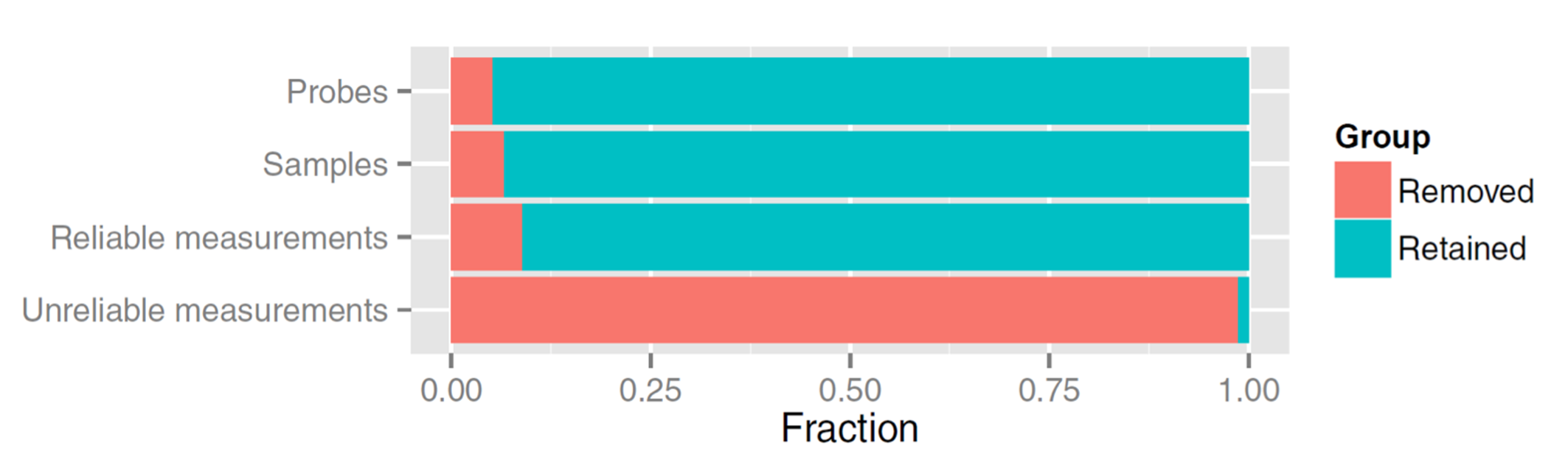

Supplement: S1 Fig — 25263 probes and 3 samples were removed from further analyses. (TIF) [file pone.0211917.s007.tif]

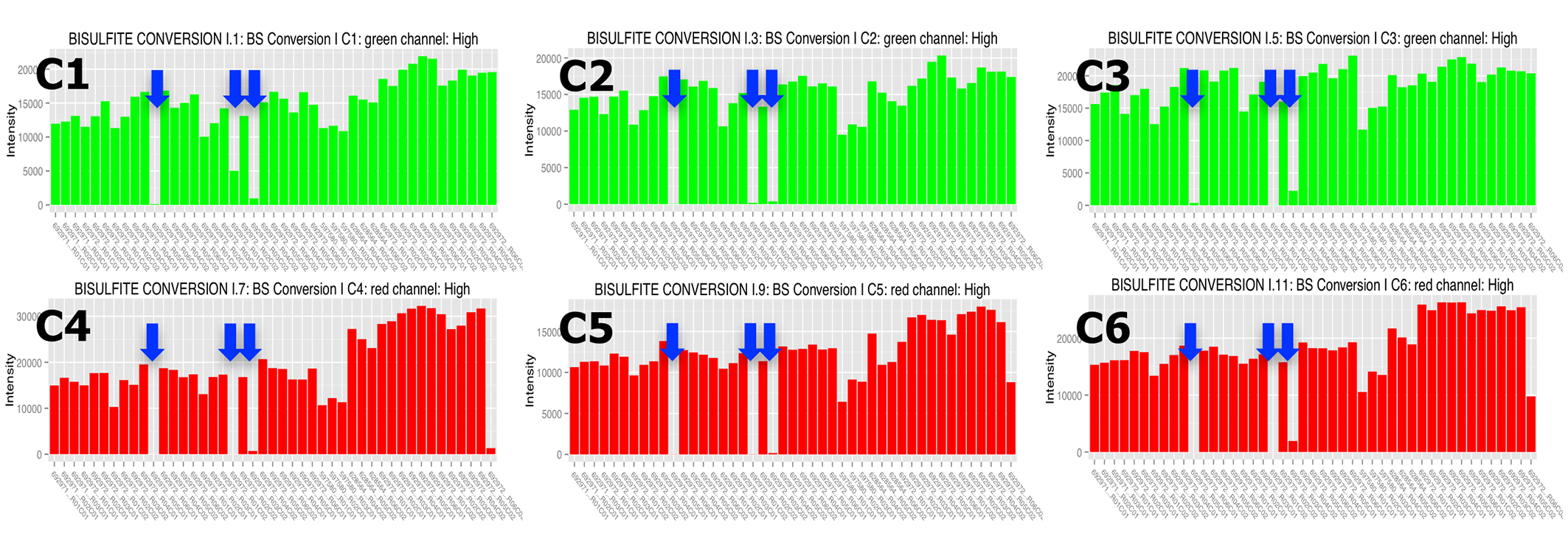

Supplement: S2 Fig — The samples indicated by the blue arrow are 6929726053_R05C01, 6929726054_R01C02 and 6929726054_R03C02 in Trio4 and Trio7, respectively. (TIF) [file pone.0211917.s008.tif]

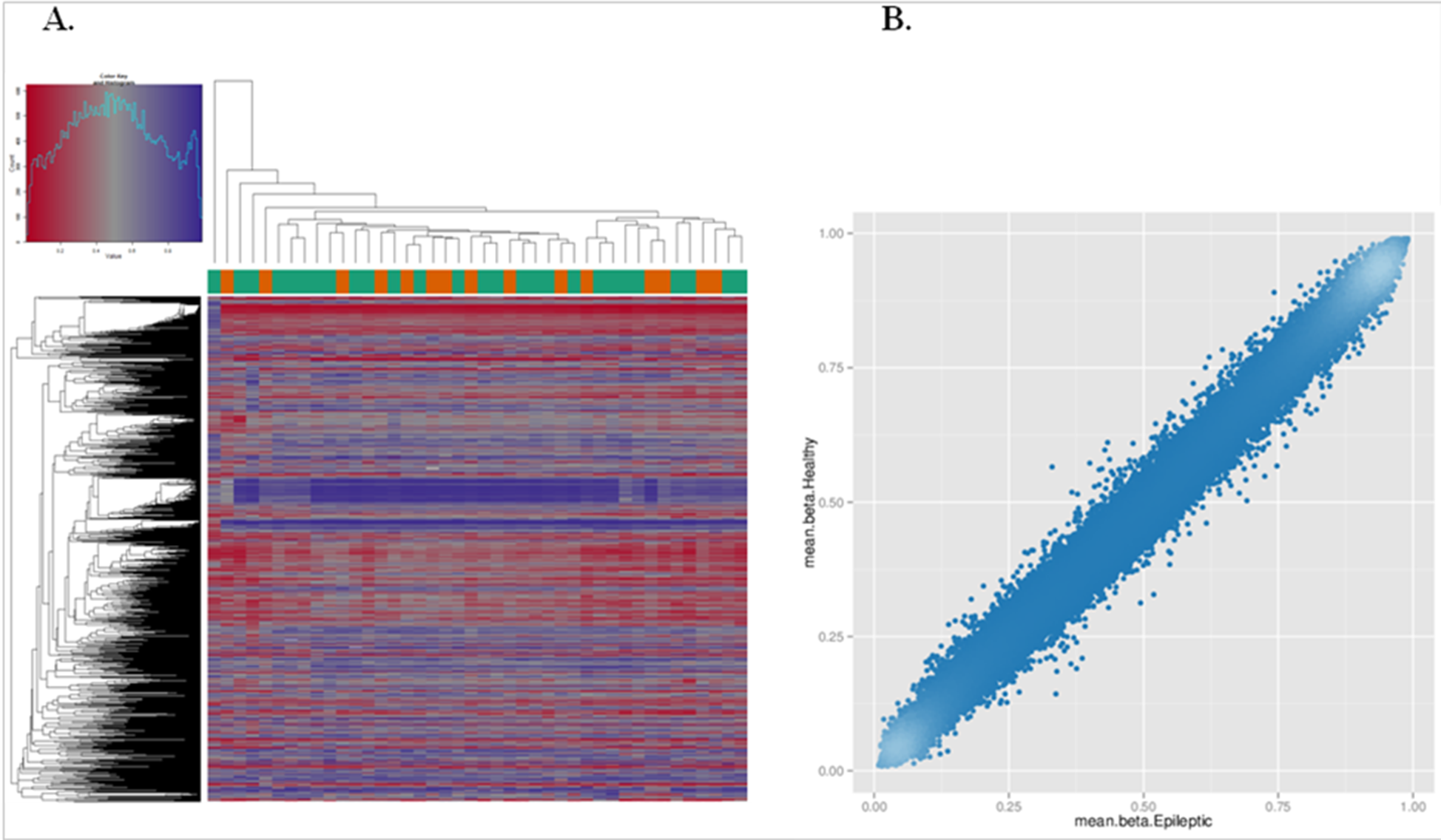

Supplement: S3 Fig — (A) Unsupervised hierarchical clustering of healthy (orange) and epileptic (green) individuals for all probes based on their beta values. The heatmap displays only CpG islands with the highest variance across all samples. Red color indicates beta values in the range of 0–0.5 (hypomethylation); blue color indicates beta values between 0.5–1 (hypermethylation) (B) Scatter plot for differential methylation analysis through T-test. The colored points (blue/red) represent differentially methylated sites according to FDR (Benjamini-Hochberg) adjustment which was set at 0.05 (None of the probes found as significant with T-test analysis after FDR adjustment, therefore there isn’t any red point on the graph). If differential methylation is not colored according to a gradient criterion, brighter colors correspond to higher point density. (TIF) [file pone.0211917.s009.tif]

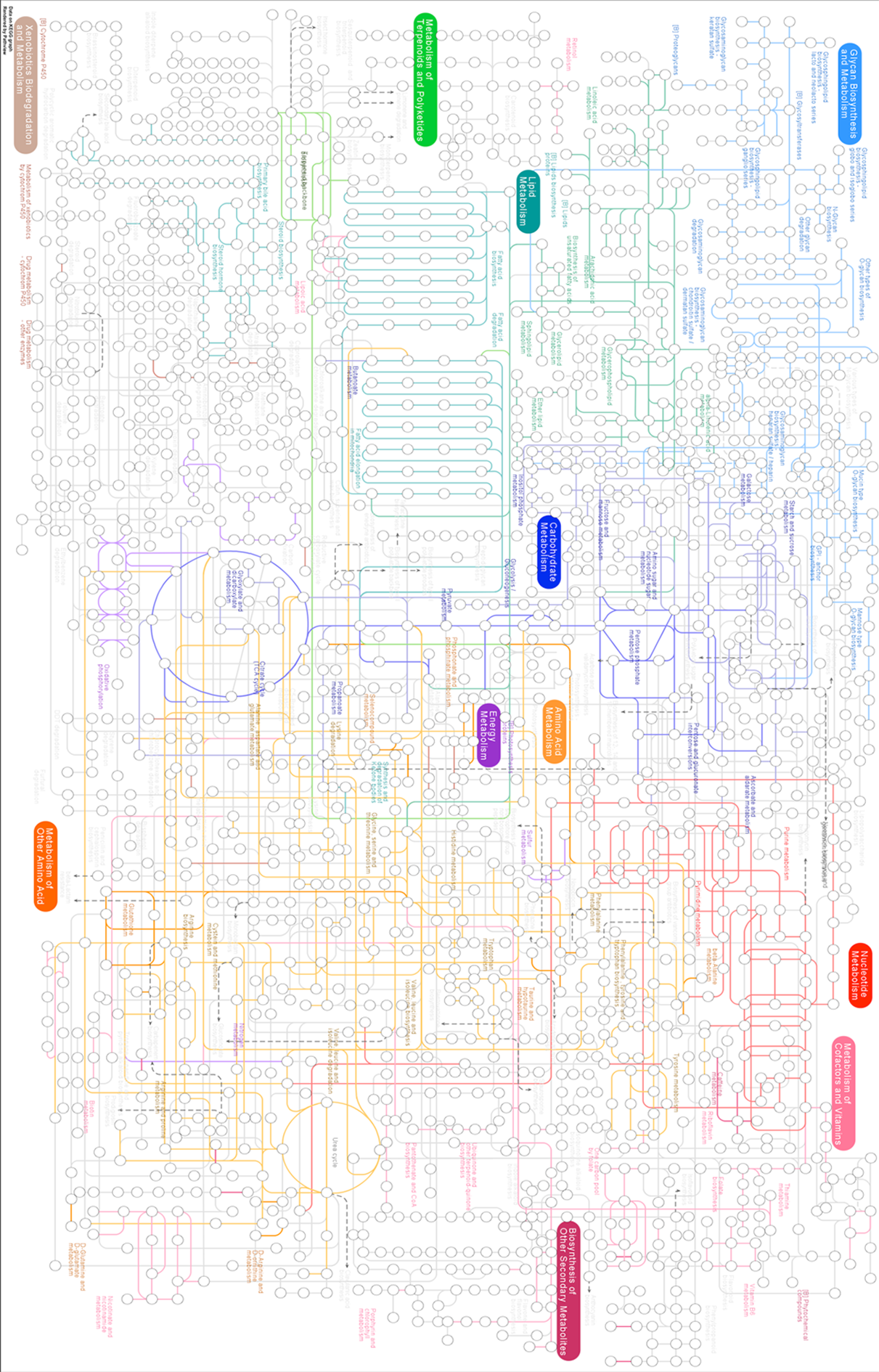

Supplement: S4 Fig — The pathway is dual-colored on the gene level using epileptic and healthy groups. Rescaled beta values are colored from green to red using the new (-1, 1) range. Epileptic group is represented on the left half and healthy group on the right half of the box representing each gene. (TIF) [file pone.0211917.s010.tif]

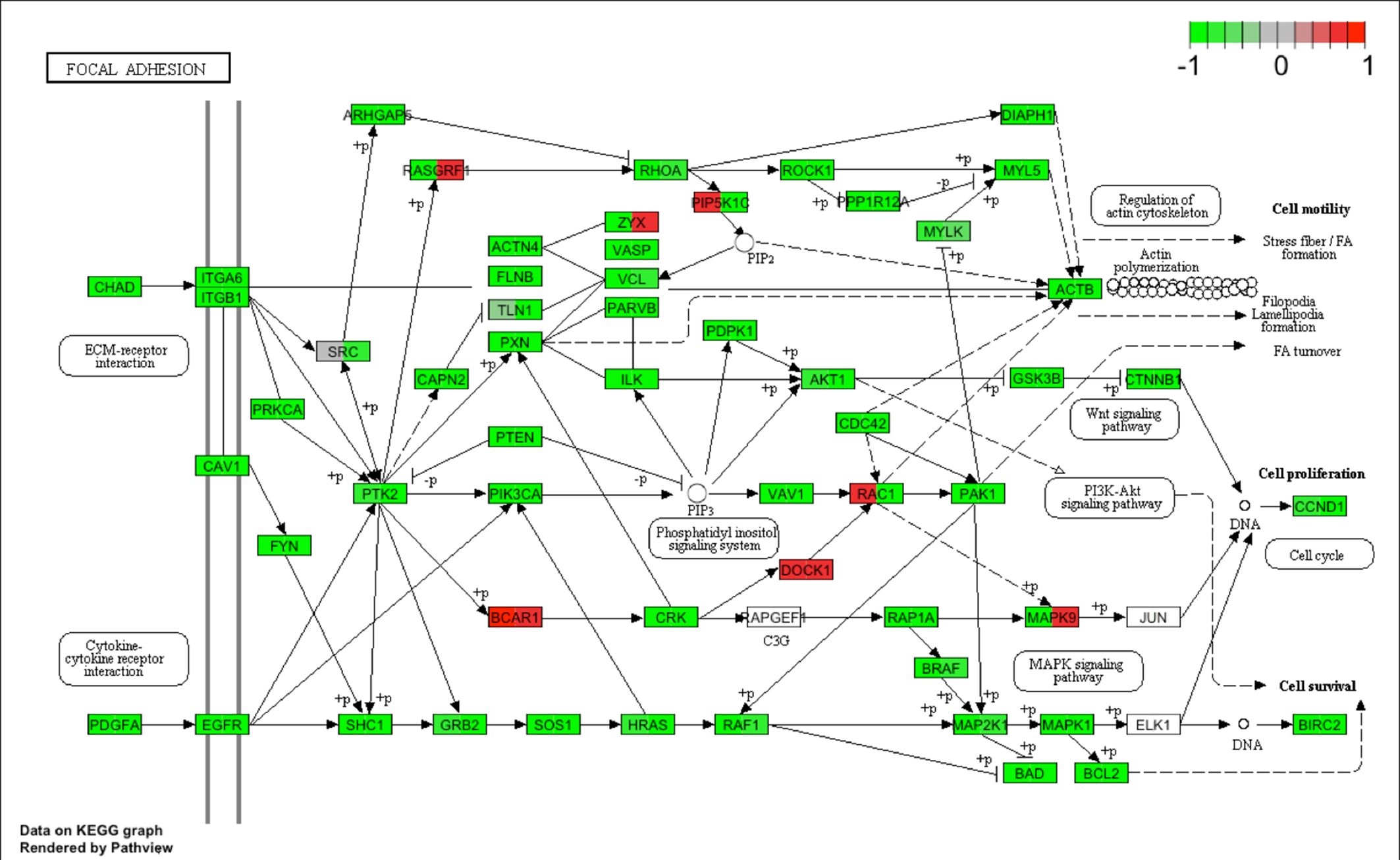

Supplement: S5 Fig — The pathway is dual-colored on the gene level using epileptic and healthy groups. Rescaled beta values are colored from green to red using the new (-1, 1) range. Epileptic group is represented on the left half and healthy group on the right half of the box representing each gene. (TIF) [file pone.0211917.s011.tif]

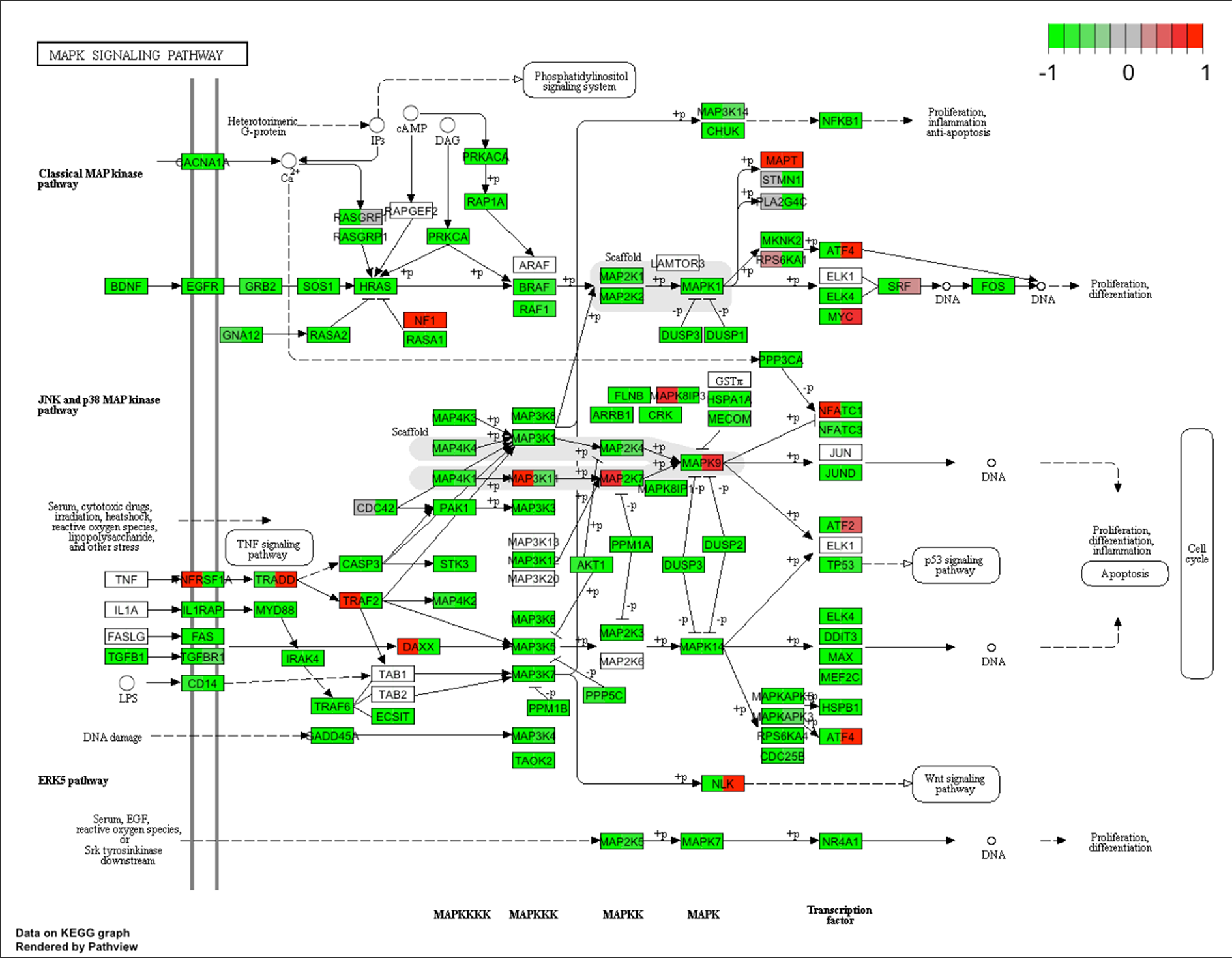

Supplement: S6 Fig — The pathway is dual-colored on the gene level using epileptic and healthy groups. Rescaled beta values are colored from green to red using the new (-1, 1) range. Epileptic group is represented on the left half and healthy group on the right half of the box representing each gene. (TIF) [file pone.0211917.s012.tif]

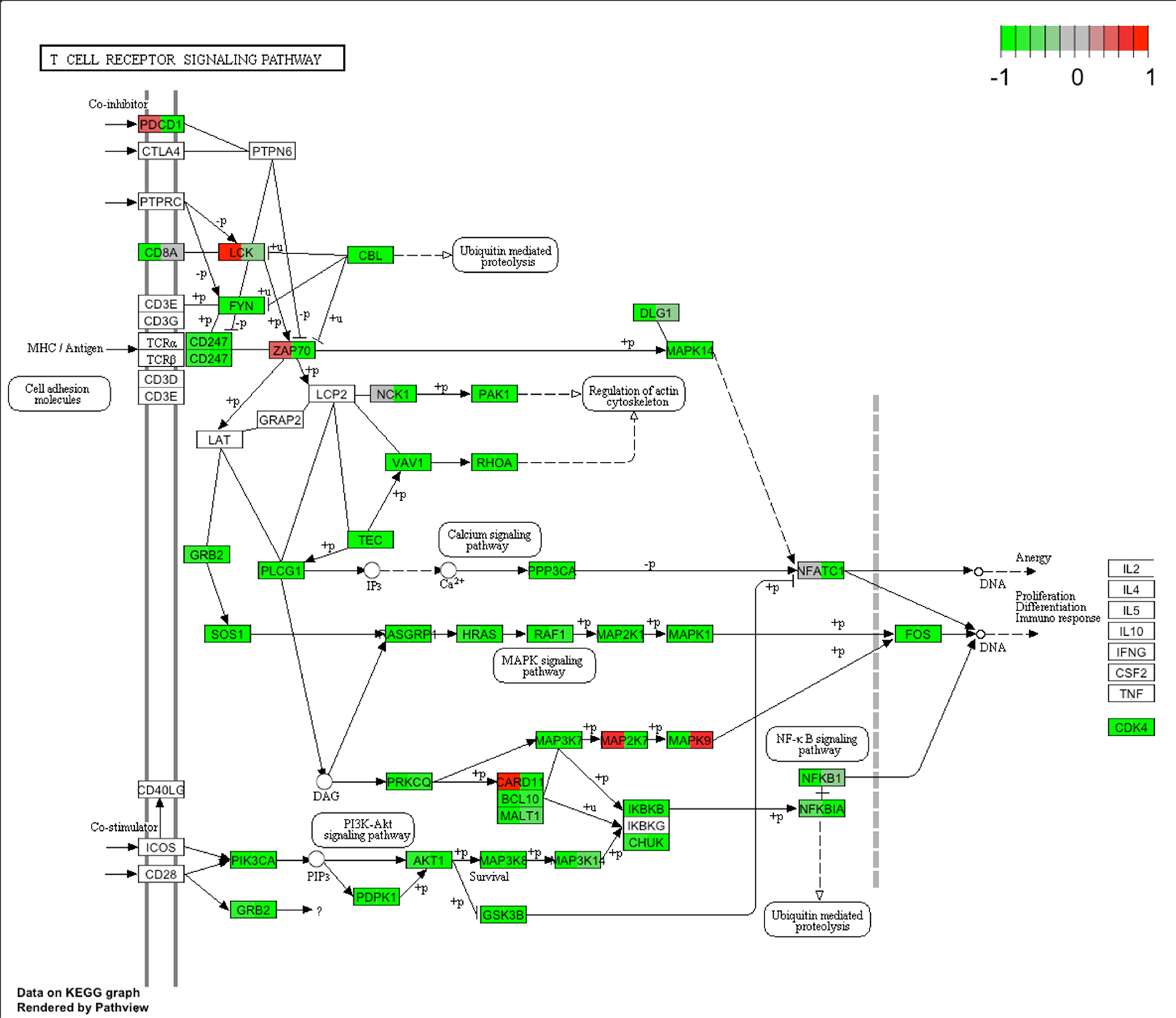

Supplement: S7 Fig — The pathway is dual-colored on the gene level using epileptic and healthy groups. Rescaled beta values are colored from green to red using the new (-1, 1) range. Epileptic group is represented on the left half and healthy group on the right half of the box representing each gene. (TIF) [file pone.0211917.s013.tif]

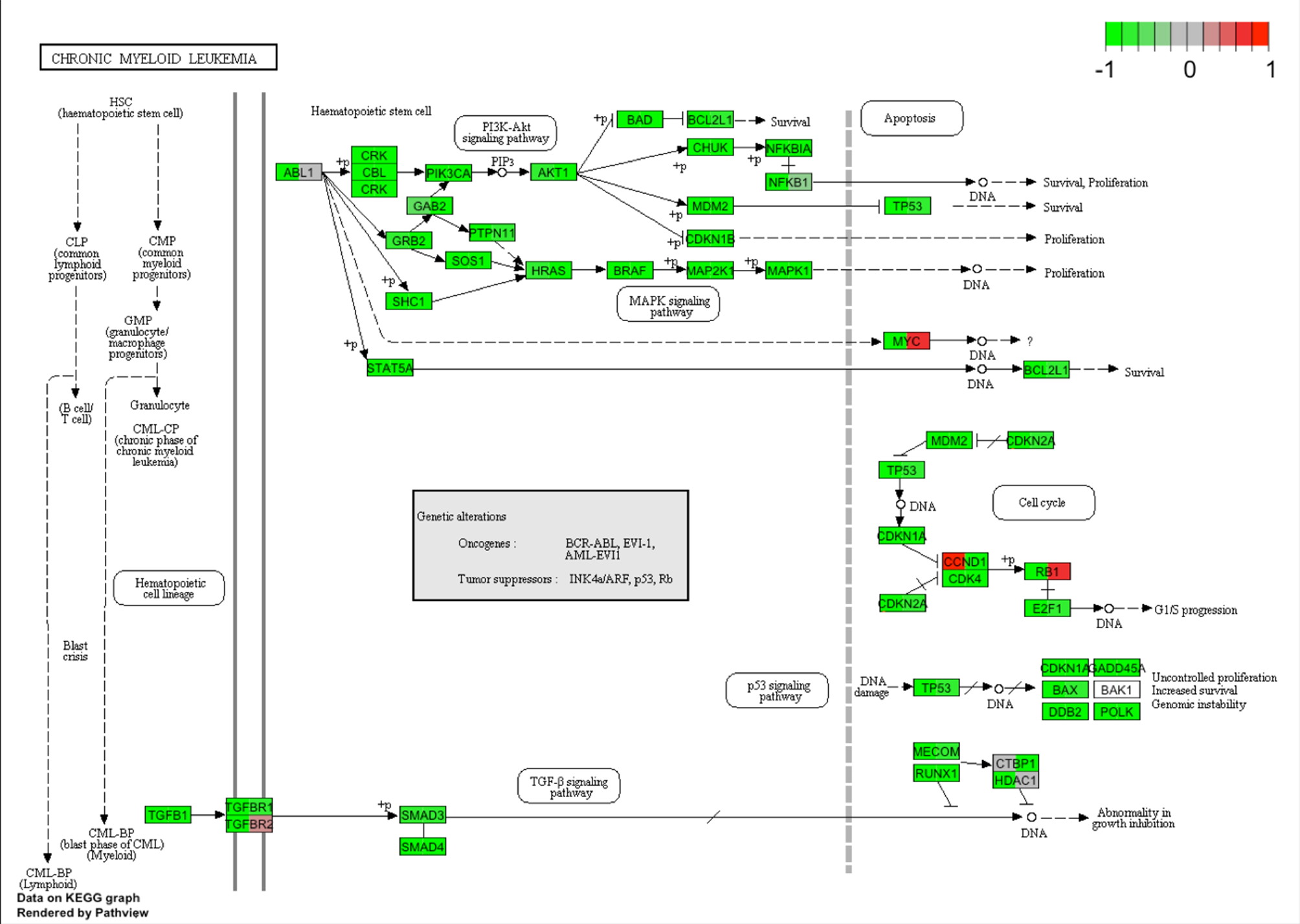

Supplement: S8 Fig — The pathway is dual-colored on the gene level using epileptic and healthy groups. Rescaled beta values are colored from green to red using the new (-1, 1) range. Epileptic group is represented on the left half and healthy group on the right half of the box representing each gene. (TIF) [file pone.0211917.s014.tif]
